# Accelerometer-Assessed Physical Activity and Sedentary Time at School for Children with Disabilities: Seasonal Variation

**DOI:** 10.3390/ijerph16173163

**Published:** 2019-08-30

**Authors:** Cindy H.P. Sit, Wendy Y. Huang, Jane J. Yu, Thomas L. McKenzie

**Affiliations:** 1 Department of Sports Science and Physical Education, The Chinese University of Hong Kong, Hong Kong, China; 2 Department of Sport and Physical Education, Hong Kong Baptist University, Hong Kong, China; 3 School of Exercise and Nutritional Sciences, San Diego State University, San Diego, CA 92120, USA

**Keywords:** seasons, children, adolescents, schools, physical education, physical activity, sedentary time

## Abstract

Schools are salient locations for children with disabilities to accrue physical activity (PA) and to diminish sedentary time (ST). We examined seasonal variation in accelerometer-assessed PA and ST among children with disabilities during the school day in three school settings (physical education (PE) lessons, recess and lunchtime). Children (*n* = 270) from 13 special schools for those with five disability types (visual impairments, hearing impairments, physical disabilities, intellectual disabilities (ID), and social development problems) participated. Their PA and ST were assessed during three winter and three summer school days using accelerometry. Linear mixed models were performed to determine seasonal variation in the proportion of time they spent in moderate-to-vigorous physical activity (MVPA) and ST in the three settings. On average, the children spent 4.5% (18.6 min) and 4.0% (15.6 min) in MVPA at school during winter and summer days, respectively. They were more physically active during winter (especially during recess and lunchtime), but there were no seasonal differences for ST. Thus, children’s year-round engagement in PA needs to be promoted, especially during summer.

## 1. Introduction

The association of physical activity (PA) with health benefits in children, regardless of their disability, is well documented [1,2]. Compared to those with typical development, children with disabilities are less physically active, engage in fewer physical recreation activities, and are at higher risk for diseases such as diabetes and obesity [3,4,5]. Studies using accelerometry have found that only a small proportion of children with disabilities meet the 60 min per day of moderate-to-vigorous physical activity (MVPA) recommendation [3,6,7].

Children’s PA and sedentary time (ST) are greatly shaped by their surrounding environments, and health entities have suggested that a major portion of their recommended MVPA time be accrued at school [8,9]. It has been recently suggested that children should accumulate at least 30 min of MVPA at school [10]. Unfortunately, schools worldwide are falling short in providing PA [11], and our recent study showed that Hong Kong children with disabilities accrued only 17 min of MVPA daily at school while spending about 70% of their time on campus being sedentary [12]. That study and others [13] clearly indicate that efforts are needed to identify factors that influence the PA and ST of special needs children so that effective interventions be designed.

Socio-ecological investigations have highlighted the importance of examining environmental influences on children’s PA and ST, including seasonal variation [14,15]. A review paper highlighted the importance of seasonal influence on children’s PA and ST and reported regional differences in seasonal variation in accelerometer-assessed PA and ST, with U.K. children found to be more active during summer than winter [15]. The review findings for studies in other European countries and the U.S. were inconclusive, but more recent studies have reported seasonal variation. For example, children in the cool and dry climates of European countries have been found to be less active and more sedentary during winter than during spring and summer [16,17], and in Australia where temperatures are high during summer, similar to Hong Kong, children’s PA was lower than during winter [18]. Subsequently, an international comparative study showed that, in general, there was a linear and positive relationship between PA and temperatures between 0 and 20 °C and that higher temperatures were associated with reduced activity levels [14]. Meanwhile, another study showed a curvilinear relationship between PA and temperatures between 20 and 22 °C [19].

One review paper examined seasonal variation in children’s PA during school time, including recess, and found the results to be inconclusive [20]. Meanwhile, a recent study found children to be less physically active during lunchtime in spring and summer than in winter but found no seasonal differences during recess [21]. All children in the aforementioned studies were of typical development. While one recent review reported winter/cold weather to be a barrier to PA among those with intellectual disabilities [22], there is a paucity of research examining seasonal variation in PA and ST among individuals with disabilities. Such studies are needed because children with disabilities typically spend a major portion of their day at school year-round and this setting may have a variety of PA opportunities including physical education (PE) lessons, recess, and after school programs. Generating detailed information could aid in the development of effective PA interventions that could cater specifically to seasonal needs, such as the design and availability of indoor spaces and activities that could support MVPA during extremely hot or cold temperatures.

To the best of our knowledge, the current study is the first to examine seasonal variation in both accelerometer-assessed PA and ST among children with diverse disabilities. Hong Kong, the location of the study, has a typical subtropical climate that is characterized by hot and humid summers (mean monthly temperature, 25–28 °C; relative humidity over 80%) and generally pleasant, sunny winters (mean monthly temperature, 15–17 °C; relative humidity, 50–70%) [23]. The purpose of the study was to examine seasonal variation in accelerometer-assessed PA and ST in children with disabilities during three different school settings (PE lessons, recess, and lunchtime).

## 2. Materials and Methods

### 2.1. Participants

Participants were recruited in 2013 from 13 Hong Kong special schools designed to accommodate the needs for children with five main disability types: Visual impairments (VI) (*n* = 1 school), hearing impairments (HI) (*n* = 1 school), physical disabilities (PD) (*n* = 1 school), intellectual disabilities (ID) (*n* = 10 schools), and social development problems (SD) (*n* = 1 school). Table 1 identifies the number of children by disability type and gender, and the details of the study design have been described elsewhere [12]. Briefly, students in grades 1–12 within each school were invited to participate and parental written consent and child assent were provided for 313 Hong Kong Chinese boys and girls. Data collection was conducted during winter (November 2013–March 2014, monthly mean temperature 17.7 °C, relative humidity 73%) and during summer (June–July 2014, monthly mean temperature 29.4 °C, relative humidity 80%). The study complied with the principles of the Declaration of Helsinki and was approved by the Joint Chinese University of Hong Kong-New Territories East Cluster Clinical Research Ethics Committee (Reference number: CRE-2013.512).

### 2.2. Measures

Participating students wore an ActiGraph accelerometer (GT3X model; ActiGraph, Pensacola, FL, America), a widely used objective measure of PA and ST with children, including those with disabilities, during three school days each season. Assessment days were not necessarily consecutive, and at least one day each season included a PE lesson.

Trained research assistants visited the schools on each measurement morning to ensure the accelerometers were attached appropriately above the child’s right hip via an elastic belt and they returned at the end of the school day to collect the instruments. Schools provided detailed schedules to identify the specific time periods that identified as before school, PE lessons, recess, lunchtime, and after school and rehabilitation sessions at school. A 15-s epoch was used to record the data and the cutoff points developed by Evenson et al. [24] and validated for children with disabilities were used [25]. Evenson cut-points were applied to classify MVPA (≥2296 counts per minute) and ST (≤100 counts per minute). For an assessment day to be considered valid, participants had to wear the accelerometer on campus for the full school day. Data were excluded if there were any missing minutes of data during the school day. For each day, MVPA and ST were derived for the whole school day and separately for each of the six possible different on-campus settings identified above. Only 5 of the 13 schools provided any before school or after school programs or any rehabilitation sessions, so separate analyses were completed for only 3 settings (PE, recess, and lunchtime).

### 2.3. Statistical Analysis

Descriptive statistics were performed to identify total accelerometer wear time as well as the duration and proportion of time spent in MVPA and ST in the three settings. Linear mixed models (LMMs) were performed to determine seasonal variation in the proportion of time students spent in MVPA and ST during the whole school day and separately in the 3 settings. LMMs were used because they accommodate repeated measures (3 summer and 3 winter days) while adjusting for school-level clustering. The models were further adjusted for sex, grade level, total accelerometer wear time, and disability type. Sex-season interactions were examined, and no interactions were found. The statistical analyses were performed using SPSS version 25.0 (IBM Corp, Armonk, USA), and the *p* value was set at 0.05.

## 3. Results

The final data analysis included 270 students (162 boys; 108 girls) in Grades 1–12 who provided valid accelerometer data for at least one school day during both winter and summer seasons (Table 1). Excluded from the analyses were 15 students who were wheelchair bound or needed assistance to walk and eight who had missing gender information. On average, participants wore the accelerometers at school 6.9 h per day during winter, spending 4.5% (18.6 min) of that time in MVPA. In contrast, they wore accelerometers for 6.5 h per day during summer, spending 4.0% (15.6 min) of the school day in MVPA (Table 2). Approximately 70% of their school day was spent being sedentary during both seasons. During both seasons, students were more active during PE than during recess and lunchtime.

Overall, the students were more physically active at school in winter than in summer (*b* = 0.39; 95% CI, 0.11 to 0.67; Table 3), but seasonal differences were not found for ST (Table 4). Table 3 and Table 4 show seasonal variation in MVPA and ST during PE lessons, recess, and lunchtime periods, with adjustments made for sex, grade level, total accelerometer wear time, and disability type. Students were more physically active in winter than in summer during both recess (*b* = 2.03; 95% CI, 1.15 to 2.91; Table 3) and lunchtime (*b* = 0.60; 95% CI, 0.09 to 1.10; Table 3). In addition, they were less sedentary in winter than in summer during PE lessons (*b* = −4.55; 95% CI, −9.00 to −0.10; Table 4) and during lunchtime (*b* = −1.75; 95% CI, −3.17 to −0.34; Table 4).

## 4. Discussion

Previous studies had not examined seasonal variation in accelerometer-assessed MVPA and ST for children with different disability types. We found that children with various disabilities were generally more active at school during winter than summer; a result consistent with a study of typically developing children [18]. The reduced summer MVPA may be due to the high temperatures and relative humidity in Hong Kong during that season. We did not find a seasonal difference for overall ST at school, and this result concurs with a review of ST studies with typically developing children [15]. In contrast, a recent study found that typically developing Japanese children had decreased PA and increased ST during hot and humid summer weather [26]. It appears that hot weather has a greater influence on children’s MVPA than ST, including those with disabilities.

One study of typically developing children also found significant sex-season interaction effects [18], with girls being more active than boys during autumn than during spring and summer. Our study with special needs children did not find such interaction effects, possibly because it was limited to two seasons. Future research that examines all four seasons is warranted in order to provide a broader picture of seasonal effects that might enhance intervention planning.

Interestingly, we found seasonal variation in MVPA and ST across PE, recess, and lunchtime after adjusting for sex, grade level, and time in the three school settings. Specifically, children with disabilities were less sedentary during PE and more active during recess and lunchtime in winter. Previous research [22] showed that typically developing children were more active during lunchtime in winter than in summer, and this was consistent with the present study. Our study also found similar patterns during PE and recess. This may be due to the need of thermoregulation to keep the body warm in winter [27]. This conjecture, however, warrants future research attention. It is common practice for children in Hong Kong schools to have both PE classes and free play opportunities held in a (covered) open playground. As Hong Kong is typically very hot and humid during the summer, it is not surprising that children were found to be less active during this season.

The recent Hong Kong 2018 report card on PA for children and youth [28] concluded that children’s low MVPA and high ST were likely affected by their immediate environment, including schools. Having access to activity-enhancing indoor facilities would be advantageous to children with disabilities, especially during summer. However, our previous study of activity areas in special school environments before and after school, recess, and lunch [29] showed that although PA areas were typically usable (92% of the time), they were accessible, provided loose equipment, and had organized activities only 47%, 33%, and 2.8% of the time, respectively. Additionally, we have also shown that children’s PA is influenced by teacher behavior and lesson context [30]. Thus, strategies to modify both the physical and social environments are needed, and these should include changes to school policies that support access to facilities and improved programming.

## 5. Conclusions

Increasing children’s PA is an important public health issue worldwide, and schools can play a significant role in promoting active behavior in children all year round [8]. The present study is the first to examine seasonal variation in both MVPA and ST of children with disabilities. The challenge is how to minimize seasonal variation and support children accumulating more PA during both structured and unstructured times at school [22].

Among the strengths of the study is its use of accelerometers to objectively measure PA and ST with children of five disability types in 13 special schools (21.7% of the special schools in Hong Kong). Study limitations include completing assessments during two seasons only and not being able to fully consider all potentially influencing contextual factors, including weather conditions beyond temperature and humidity (e.g., air pollution, solar radiation, and rainfall). Generating additional detail on contextual conditions could possibly better inform interventions. As well, the study was conducted in Hong Kong where the schools were designed specifically for children with specific disabilities; thus, the findings may not be generalizable to children with disabilities enrolled in inclusive/mainstreamed schools. Nevertheless, the results suggest the need for school policymakers and teachers to consider infrastructure and programmatic designs that will promote opportunities of children with disabilities to engage in PA, especially during seasons with high temperatures and relative humidity.

## Figures and Tables

**Table 1:** Number of children by disability type and sex (total *n* = 270 with valid data during both winter and summer).

Type of Special School	Female	Male	Total
Visual impairments	10	11	21
Hearing impairments	5	6	11
Physical disabilities	12	12	24
Intellectual disabilities (mild)	42	69	111
Intellectual disabilities (moderate)	23	35	58
Intellectual disabilities (severe)	16	17	33
Social development problems	0	12	12

**Table 2:** Time spent in MVPA and ST overall and during 3 segments of the school day in both seasons.

Time Segment	*n*	Winter			Summer		
		Duration (min)	% MVPA	% ST	Duration (min)	% MVPA	% ST
Wearing time	270	412.4 (39.2)	4.5 (2.8)	68.9 (11.5)	390.9 (30.9)	4.0 (2.5)	70.0 (10.9)
PE lessons	134	55.1 (17.1)	13.9 (11.3)	48.5 (21.3)	55.1 (20.8)	12.5 (9.9)	51.0 (21.0)
Recess	256	33.5 (12.0)	9.4 (9.1)	51.7 (37.6)	32.7 (17.3)	7.1 (6.8)	56.6 (18.0)
Lunchtime	262	63.8 (12.9)	4.5 (5.0)	68.2 (15.2)	66.8 (13.5)	3.9 (4.0)	69.0 (14.8)

Notes: Data are shown in mean (SD). PE, physical education; MVPA, moderate-to-vigorous physical activity; ST, sedentary time; SD, standard deviation.

**Table 3:** Seasonal variation in MVPA (% of wear time) at school, during PE lessons, recess, and lunchtime.

Variable	At School		During PE Lessons		During Recess		During Lunchtime	
	Coefficient	95% CI	Coefficient	95% CI	Coefficient	95% CI	Coefficient	95% CI
Season (reference: Summer)	**0.39**	**0.11, 0.67**	0.63	−1.74, 3.00	**2.03**	**1.15, 2.91**	**0.60**	**0.09, 1.10**
Sex (reference: Boys)	**−1.43**	**−1.94, −0.93**	−1.76	−3.64, 0.12	**−3.17**	**−4.68, −1.66**	**−1.84**	**−2.63, −1.06**
Grade level (range, 1–12)	−0.01	−0.08, 0.05	**0.35**	**0.07, 0.62**	0.06	−0.14, 0.26	**0.11**	**0.00, 0.22**
Total wear time (min/day)	0.00	−0.00, 0.00	**−0.13**	**−0.20, −0.06**	−0.01	−0.06, 0.04	**0.05**	**0.01, 0.09**

Notes: MVPA, moderate-to-vigorous physical activity; PE, physical education; CI, confidence interval. Findings are presented as regression coefficients and 95% confidence intervals and were based on linear mixed models with school as random effects, and seasons and assessment days as repeated-measure variables. The models were adjusted for sex, grade level, total wearing time and disability type. Statistically significant results are in bold.

**Table 4:** Seasonal variation in ST (% of wear time) at school, during PE lessons, recess, and lunchtime.

Variable	At School		During PE Lessons		During Recess		During Lunchtime	
	Coefficient	95% CI	Coefficient	95% CI	Coefficient	95% CI	Coefficient	95% CI
Season (reference: Summer)	−0.33	−1.38, 0.72	**−4.55**	**−9.00, −0.10**	−4.20	−9.44, 1.04	**−1.75**	**−3.17, −0.34**
Sex (reference: Boys)	**3.86**	**1.67, 6.05**	4.72	0.21, 9.24	**6.45**	**1.10, 11.80**	**4.54**	**1.58, 7.51**
Grade level (range, 1–12)	**0.95**	**0.66, 1.23**	0.35	−0.28, 0.99	0.67	−0.04, 1.38	0.04	−0.35, 0.43
Total wear time (min/day)	−0.03	−0.04, −0.02	0.20	−0.06, 0.35	−0.02	−0.25, 0.30	**−0.16**	**−0.28, −0.04**

Notes: ST, sedentary time; PE, physical education; CI, confidence interval; Findings are presented as regression coefficients and 95% confidence intervals and were based on linear mixed models with school as random effects, and seasons and assessment days as repeated-measure variables. The models were adjusted for sex, grade level, total wearing time and disability type. Statistically significant results are in bold.

## References

[1] Piercy K.L., Troiano R.P., Ballard R.M., Carlson S.A., Fulton J.E., Galuska D.A. (2018). The physical activity guidelines for Americans. J. Am. Med. Assoc..

[2] World Health Organization Global Recommendations on Physical Activity for Health 2010. https://www.who.int/dietphysicalactivity/global-PA-recs-2010.pdf.

[3] Lobenius-Palmer K., Sjoqvist B., Hurtig-Wennlof A., Lundqvist L.O. (2018). Accelerometer-assessed physical activity and sedentary time in youth with disabilities. Adapt. Phys. Activ. Q..

[4] Neter J.E., Schokker D.F., Jong E., Renders C.M., Seidell J.C., Visscher T.L.S. (2011). The prevalence of overweight and obesity and its determinants in children with and without disabilities. J. Pediatr..

[5] Woodmansee C., Hahne A., Imms C., Shields N. (2016). Comparing participation in physical recreation activities between children with disability and children with typical development: A secondary analysis of matched data. Res. Dev. Disabil..

[6] Einarsson I.T., Olafsson A., Hinriksdottir G., Johannsson E., Daly D., Arngrimsson S.A. (2015). Differences in physical activity among youth with and without intellectual disability. Med. Sci. Sports Exerc..

[7] Mitchell L.E., Ziviani J., Boyd R.N. (2015). Characteristics associated with physical activity among independently ambulant children and adolescents with unilateral cerebral palsy. Dev. Med. Child. Neurol..

[8] Centers for Disease Control and Prevention (2013). Comprehensive School Physical Activity Programs: A Guide for Schools.

[9] Institute of Medicine (2013). Educating the Student Body: Taking Physical Activity and Physical Education to School.

[10] Schranz N., Glennon V., Evans J., Gomersall S., Hardy L., Hesketh K.D. (2018). Results from Australia’s 2018 report card on physical activity for children and youth. J. Phys. Act. Health.

[11] Aubert S., Barnes J.D., Abdeta C., Nader P.A., Adeniyi A.F., Aguilar-Farias N. (2018). Global matrix 3.0 physical activity report card grades for children and youth: Results and analysis from 49 countries. J. Phys. Act. Health.

[12] Sit C.H.P., Mckenzie T.L., Cerin E., Chow B.C., Huang W.Y., Yu J. (2017). Physical activity and sedentary time among children with disabilities at school. Med. Sci. Sports Exerc..

[13] Rimmer J.H., Marques A.C. (2012). Physical activity for people with disabilities. Lancet.

[14] Harrison F., Goodman A., Sluijs E.M.F., Andersen L.B., Cardon G., Davey R. (2017). Weather and children’s physical activity: How and why do relationships vary between countries?. Int. J. Behav. Nutr. Phys. Act..

[15] Rich C., Griffiths L.J., Dezateux C. (2012). Seasonal variation in accelerometer-determined sedentary behaviour and physical activity in children: A review. Int. J. Behav. Nutr. Phy..

[16] Gracia-Marco L., Ortega F.B., Ruiz J.R., Williams C.A., Hagstromer M., Manios Y. (2013). Seasonal variation in physical activity and sedentary time in different European regions. J. Sports Sci..

[17] Hjorth M.F., Chaput J.P., Michaelsen K., Astrup A., Tetens I., Sjodin A. (2013). Seasonal variation in objectively measured physical activity, sedentary time, cardio-respiratory fitness and sleep duration among 8–11 year-old Danish children: A repeated-measures study. BMC Public Health.

[18] Ridgers N.D., Salmon J., Timperio A. (2015). Too hot to move? Objectively assessed seasonal changes in Australian children’s physical activity. Int. J. Behav. Nutr. Phys. Act..

[19] Remmers T., Thijs C., Timperop A., Salmon J., Veitch J., Kremers S.P. (2017). Daily weather and children’s physical activity patterns. Med. Sci. Sports Exerc..

[20] Ridgers N.D., Salmon J., Parrish A.M., Stanley R.M., Okely A.D. (2012). Physical activity during recess: A systematic review. Am. J. Prev. Med..

[21] Ridgers N.D., Salmon J., Timperio A. (2018). Seasonal changes in physical activity during school recess and lunchtime among Australian children. J. Sports. Sci..

[22] Bossink L.W.M., Putten A.A.J., Vlaskamp C. (2017). Understanding low levels of physical activity in people with intellectual disabilities: A systematic review to identify barriers and facilitators. Res. Dev. Disabil..

[23] Climate of Hong Kong Hong Kong Special Administrative Region, China: Hong Kong Observatory. http://www.weather.gov.hk/cis/climahk_e.htm.

[24] Evenson K.R., Catellier D.J., Gill K., Ondrak K.S., McMurray R.G. (2008). Calibration of two objective measures of physical activity for children. J. Sports Sci..

[25] Capio C.M., Sit C.H., Abernethy B.A., Eguia K.F., Masters R.S. (2015). Fundamental movement skills training to promote physical activity in children with and without disability: A pilot study. J. Sports Health Sci..

[26] Tanaka C., Reilly J.J., Tanaka M., Tanaka S. (2016). Seasonal changes in objectively measured sedentary behavior and physical activity in Japanese primary school children. BMC Public Health.

[27] Ridgers N.D., Stratton G., Fairclough S.J. (2006). Physical activity levels of children during school playtime. Sports Med..

[28] Huang W.Y., Wong S.H.S., Sit C.H.P., Wong M.C.S., Sum R.K.W., Wong S.W.S. (2019). Results from the Hong Kong’s 2018 report card on physical activity for children and youth. J. Exerc. Sci. Fit..

[29] Sit C.H., McKenzie T.L., Cerin E., McManus A., Lian J. (2013). Physical activity for children in special school environment. Hong Kong Med. J..

[30] Sit C.H., McManus A.M., McKenzie T.L., Lian J. (2007). Physical activity levels of children in special schools. Prev. Med..

